# Bacterial Isolates From Orthopedic Posttraumatic Surgical Infections in Northern Ghana: ES*β*L Detection and Analysis of Antimicrobial Resistance Trends

**DOI:** 10.1155/ijm/9949633

**Published:** 2025-08-07

**Authors:** Fredrick Gyilbagr, Williams Walana, Alexis D. B. Buunaaim, Ezekiel Kofi Vicar, Jacob Nii Otinkorang Ankrah, Emmanuel Antwi Boasiako Frimpong, Rukaya Baanah Alhassan, Ibrahim Sibdow Baako, Alahaman Nana Boakye, Samuel Addo Akwetey, Akosua Bonsu Karikari, Gideon Kofi Helegbe, Stephen Tabiri

**Affiliations:** ^1^Department of Clinical Microbiology, University for Development Studies, Tamale, Ghana; ^2^Department of Laboratory Service, Tamale Teaching Hospital, Tamale, Ghana; ^3^Department of Surgery, University for Development Studies, Tamale, Ghana; ^4^Department of Trauma Orthopedics, Tamale Teaching Hospital, Tamale, Ghana; ^5^Department of Biochemistry and Molecular Medicine, University for Development Studies, Tamale, Ghana

**Keywords:** antimicrobial resistance, bacterial profile, ES*β*L characterization, orthopedic surgery, surgical site infection

## Abstract

**Background:** Surgical site infections (SSIs) remain a critical challenge globally and are aggravated by rising antimicrobial resistance (AMR). Here, we evaluated the bacterial profile, AMR patterns, and ES*β*L characterization of isolates from patients diagnosed with SSI after trauma orthopedic surgery.

**Methods:** This prospective study was carried out at Tamale Teaching Hospital from September 2023 to May 2024. Patients were asked to provide demographic data. Samples were also collected from patients suspected of SSI and cultured for bacterial isolation, identification, and AMR characterization.

**Results:** In all, 210 patients were recruited for this study, and 14 (6.7%) out of 19 suspected cases developed SSI. Of 19 specimens, 14 (73.68%) were culture-positive, yielding 22 isolates. Monomicrobial growth were 7 (50.0%) and polymicrobial growth 7 (50.0%). Among the isolates, 3 (13.64%) were Gram-positive and 19 (86.36%) were Gram-negative bacilli. *Pseudomonas aeruginosa* (5, 22.73%) were the most common isolates, followed by *Klebsiella* spp. (4, 18.18%). ES*β*L-positive isolates were 3 (23.08%). PCR confirmed the expression of CTXM and SHV genes by two *Klebsiella* spp. and the CTXM gene by *Proteus vulgaris*.

**Conclusion:** Gram-negative bacteria, particularly *Pseudomonas aeruginosa*, were the dominant isolates from surgical sites after trauma orthopedic surgery. Among the Gram-positives, *Staphylococcus aureus* was dominant. Among the Enterobacterales isolates, ESBL production was detected in three cases (23.08%), with two *Klebsiella* spp. harboring CTXM and SHV resistance genes, and CTXM in one *Proteus vulgaris*. The current study has revealed varied resistant patterns of AMR, with CTXM and SHV as common ES*β*L genes among the isolates. The clinical identification of CTX-M and SHV genes could guide clinicians to consider alternative treatments to optimize therapeutic outcomes and limit the spread of resistant pathogens.

**Summary**

• The research found a 6.7% incidence rate of surgical site infections (SSIs) among patients undergoing trauma-related orthopedic surgeries in Northern Ghana. Gram-negative bacteria (86.36%) were the dominant isolates from surgical sites in the study area.
• Among the Gram-negative bacilli isolated, *Pseudomonas aeruginosa* was the most prevalent pathogen, accounting for 26.32% (five cases), followed by *Klebsiella* species at 21.05% (four cases), as the leading contributors to SSIs associated with trauma-related orthopedic procedures in Northern Ghana.
• ES*β*L production was seen among three (23.08%) isolates of the Enterobacterales family, involving CTXM and SHV genes in two *Klebsiella* spp. and CTXM in one *Proteus vulgaris*.

## 1. Introduction

SSIs are among the most frequent aftereffects of orthopedic surgery and continue to be a significant global concern for patients who have undergone any orthopedic surgery [1]. An orthopedic surgical site infection (OSSI) refers to an infection that develops within 30 days after surgery or 1 year if the surgical procedure involved the placement of an implant [2]. In orthopedic surgery, SSIs can lead to a range of clinical, economic, and societal ramifications, such as extended hospital stay, elevated rates of morbidity and mortality, heightened risk of readmission and reoperation, high cost of treatment, and undesirable outcomes [3, 4]. The risk of developing an infection following implant fixation is predicted to be between 0.5% and 30% (0.5%–2% in the case of closed fractures and up to 30% in the case of open fractures) [5]. A multicentered cohort study reported an incidence of SSI between 3.9% and 8.0% [6]. The infection can manifest as superficial SSI and deep SSI involving the underlying bone or the implant [1].

One of the primary challenges in managing SSIs is the growing issue of antimicrobial resistance (AMR). Various epidemiological influences shape the types of bacteria involved in these infections. Among the most commonly identified pathogens in SSIs are *Staphylococcus aureus*, coagulase-negative *Staphylococcus* species, *P. aeruginosa*, *Enterococcus*, *Acinetobacter*, *Klebsiella*, *Escherichia coli*, and *Proteus* species [5, 7]. These pathogens have varying degrees of resistance premised on their global distribution. The escalating global incidence of antibiotic-resistant organisms can be attributed to various reasons, such as social determinants, economic factors, healthcare services, governance, and environmental variables [8, 9].

A major group of bacterial agents of concern in AMR is extended-spectrum beta-lactamase (ES*β*L)–producing agents. ES*β*Ls are enzymes produced by bacteria that degrade *β*-lactam antibiotics with extended spectrum. In recent studies, more than 500 beta-lactamases have been identified [10, 11]. These beta-lactamases are widely distributed worldwide and are primarily found in Gram-negative bacteria, particularly those belonging to the CTX-M-15 family, which have a common resistance mechanism mediated via plasmid or produced chromosomally. Over 120 different types of ES*β*L enzymes are often found in members of the Enterobacterales family. ES*β*Ls are primarily obtained through horizontal gene transfer and offer resistance against oxyimino-cephalosporins [12]. A subset of these enzymes, known as mutant derivatives of established plasmid-mediated *β*-lactamases (TEM/SHV) or mobilized from environmental bacteria (CTX-M), hydrolyzes penicillin, monobactams, and broad-spectrum cephalosporins [13]. They are inhibited by clavulanic acid; however, they are unaffected by cephamycin and carbapenems. Clinicians involved in treating infectious diseases resulting from SSI appreciate the enormous expansion and diversity of *β*-lactamases as a risk to treatment failure. Clinically, identifying these resistant genes serves as a predictor of AMR and a means to inform antibiotic choice for meaningful therapeutic outcomes. Research indicates a rising trend of AMR in Ghana, with multidrug resistance (MDR) increasingly observed among Gram-negative bacteria, particularly in clinical and hospital settings [14, 15]. Earlier prospective studies have identified notably high levels of ESBL-producing Enterobacterales in both colonization (carriage) and clinical infection samples. This suggests individuals can harbor these resistant bacteria asymptomatically, serving as reservoirs for potential transmission and subsequent infections, especially in healthcare settings [16]. ESBL-positive bacteria, particularly those producing CTX-M enzymes, resist most *β*-lactam antibiotics. These strains are also frequently associated with coresistance to other major antibiotic classes, including aminoglycosides, fluoroquinolones, sulfonamides, and tetracyclines [17, 18]. This MDR significantly limits treatment options and underscores the importance of robust antimicrobial stewardship and surveillance efforts. This study investigated the AMR pattern and characterized ES*β*L in bacterial isolates from SSIs post trauma orthopedic surgery (TOS) in Northern Ghana.

## 2. Methodology

### 2.1. Ethical Consideration

The study was granted ethical clearance by the Institutional Review Board of the University for Development Studies (reference: UDS/IRB/127/23). Authorization to conduct the research was obtained from both the Tamale Teaching Hospital (TTH) and its surgery department (reference: TTH/R&B/SR/283). Participation in the study was entirely voluntary, with informed consent duly provided by all participants.

### 2.2. Study Area

The research was conducted at TTH, in Tamale, the capital of Ghana's Northern Region. As a major referral center for the Northern, Savanna, North East, Upper East, and Upper West regions, TTH was selected due to its high volume of orthopedic cases, diverse patient demographics, and the anticipated rise in trauma-related admissions linked to increased motorized transport in the area. With a capacity of 800 beds, the hospital provides a wide range of services from general practice to specialist services including psychiatry. It is staffed by experienced healthcare professionals and administrative teams. Beyond clinical care, TTH is also a hub for medical research, contributing to advancements in health knowledge and service delivery.

### 2.3. Study Design and Inclusion and Exclusion Criteria

This prospective study was conducted at the TTH between September 2023 and June 2024. Postoperative patients were monitored for SSIs over a follow-up period ranging from 6 to 10 months. The study population included individuals who underwent surgery and were admitted to the trauma and orthopedic surgical ward. Exclusion criteria were applied to patients admitted without undergoing surgery, those with preoperative wound infections confirmed by both clinical diagnosis and positive culture results, and individuals with incomplete medical records, specifically those who discontinued participation within 6 months and for whom no SSI had been documented before withdrawal.

### 2.4. Sample Collection for Suspected SSI

After the operation, each patient was monitored and followed within 6 weeks, 3 months, and 6 months for SSI. The patients were scheduled for periodic reviews where the surgeon examined them for the progress of wound healing and the development of SSI. The determination of SSI was based on the clinical examination and diagnosis by the surgeon according to the CDC (2013) criteria for diagnosing SSIs. Once the surgeon confirms the diagnosis, specimens are aseptically collected for bacterial culture. Nineteen samples (17 superficial and two deep wound swabs) were collected. The labeled specimens are transported in Stuart media within 1 h to the Microbiology Laboratory Department at TTH for culture, antibiogram, and Ziehl–Neelsen (ZN) staining. Bacterial suspensions were sent to the Postgraduate Research Laboratory of the School of Medicine of the University for Development Studies for genomic DNA isolation and PCR amplification of targeted ES*β*L-associated genes.

### 2.5. Culture and Sensitivity Testing

Using standard bacteriological procedures, bacterial cultures were done on blood agar, chocolate agar, and MacConkey agar. Cultures on chocolate agar were incubated at 35°C–37°C in 5% carbon dioxide (for microaerophiles), while those on blood agar and MacConkey agar were incubated at 35°C–37°C in ambient air (for facultative anaerobes and obligate aerobes) for 18–24 h. After culturing, Gram stain was performed on distinct colonies to categorize the isolates into Gram-positive and Gram-negative bacteria to inform downstream investigations.

### 2.6. ZN Staining

The ZN staining was done on all the specimens for *Mycobacterium* species identification.

### 2.7. Biochemical Test

For identification, biochemical tests, such as catalase and coagulase tests, were performed on Gram-positive organisms. In contrast, the urea test, citrate test, triple sugar iron test, motility test, oxidase test, and indole test were performed on all Gram-negative isolates.

### 2.8. Antimicrobial Susceptibility Testing (Disc Diffusion)

Antibiotic susceptibility testing was done using Kirby–Bauer's disc diffusion method, and the results were interpreted according to the Clinical and Laboratory Standards Institute (CLSI) guidelines (2022).

### 2.9. Phenotypic ES*β*L Screening

A phenotypic confirmatory disc diffusion test was used for ES*β*L screening. Mueller Hinton (MH) agar was inoculated with a standard inoculum (0.5 McFarland) of the test isolate. It was tested for ceftazidime (30 *μ*g) and ceftazidime-clavulanic acid (30 *μ*g/10 *μ*g). An increase in zone diameter of ≥ 5 mm in the presence of clavulanic acid compared to ceftazidime alone was interpreted as an ES*β*L producer.

### 2.10. DNA Extraction From Bacterial Isolates

Bacteria were collected from culture suspensions by centrifugation and 200 *μ*L dispensed into 250 *μ*L of potassium-solubilizing bacteria (KSB), according to the kit manufacturer's guide (KH Medical, Germany). A 20 *μ*L of proteinase K (100 *μ*g/mL) was added and 5 *μ*L of carrier molecule followed, vortexed for 10 s, and incubated at 56°C for 10 min. After adding 359 *μ*L of absolute ethanol and vortexing for 10 s, the content was transferred into a spin column, centrifuged at 12,000*g* for 1 min, and the flow through was discarded. A 500 *μ*L of KSW1 was added to the column and centrifuged for 1 min at 12,000*g*, discarding the flow through. This was repeated with a 500 *μ*L of KSW2. A 70 *μ*L of KSE buffer was added to the spin column, incubated for 1 min at room temperature, and centrifuged for 1 min at 12,000*g* to elute the DNA. The extracted DNA was assessed for purity and concentration (Chennai Technologies, India), 1.8–2.0 purity range, and 20–50 ng concentration range, before being used for PCR.

### 2.11. PCR Amplification of ESBL Genes and Electrophoresis

Well-labeled PCR tubes were dispensed with 12.5-*μ*L PCR master mix, 0.5 *μ*L each of forward and reverse primers, and 50 ng of DNA template added and then topped up with nuclease-free water to make a total reaction volume of 25 *μ*L. The mixture was run at the following PCR conditions: denaturation at 94°C for 3 s and then 30 s, annealing at 60°C for 30 s, extension at 72°C for 30 s, and the cycle repeated 40 times. Band separation of amplicons was done on 1 1% agarose gel in TAE running buffer at 90 V and 80 A for 30 min, and images of bands were captured.

### 2.12. Definition of SSI Classifications

The SSI was classified according to the CDC. The CDC classifies SSIs into three groups [19]. These include the following.

Superficial incisional SSI: This occurs within 30 days postsurgery and affects only the skin or subcutaneous tissue at the incision site.

Deep incisional SSI: This develops within 30 days if no implant is used or within 1 year if an implant is present and the infection is linked to the procedure. It involves deeper soft tissues such as muscle and fascia.

Organ/space SSI: This also arises within 30 days (or up to 1 year with an implant) and involves any anatomical area—excluding the incision—that was accessed or manipulated during surgery, such as internal organs or body cavities.

### 2.13. Statistical Analysis

The data were analyzed using GraphPad Prism Version 8. Frequencies, percentages, and cross-tabulations were used to summarize the demographic variables, ES*β*L screening panel, and the target genes used to identify AMR. We utilized heat maps and bar graphs to demonstrate the antimicrobial-resistant patterns against the respective organisms. Furthermore, Microsoft imaging techniques were employed to outline the various ES*β*L variants identified against their respective organisms that expressed those genes.

## 3. Results

### 3.1. Sociodemographic Characteristics and Incidence of SSIs Among the Study Participants

The study enrolled 210 patients, ranging in age from 8 months (0.67 years) to 86 years. The median age was 31.5 years, with an interquartile range of 18–47 years. The most represented age brackets were 21–30 (18.6%) and 41–50 (19.0%). Males comprised the majority of participants (68.6%), and over half were married (55.7%). Regarding residence, 42.4% lived in rural areas, 36.7% in urban settings, and 21.0% in periurban communities. SSIs were identified in 14 patients, accounting for 6.7% of those who underwent TOS.

### 3.2. Bacterial Profile and Antimicrobial-Resistant Patterns

**Figure 1:**
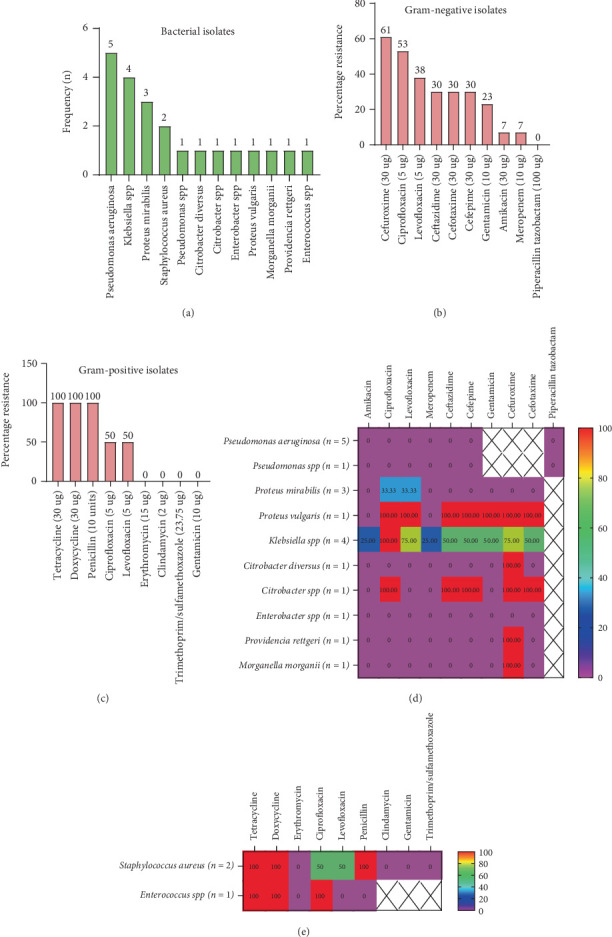
Bacterial profile and antimicrobial-resistant patterns. (a) All bacteria isolates. (b) Gram-negative bacterial isolates and pattern of AMR. (c) Gram-positive bacterial isolates and pattern of AMR. (d) Heatmap presentation of antibiotic resistance pattern among the Gram-negative isolates. (e) Heatmap presentation of antibiotic resistance pattern among the Gram-positive isolates.

A total of 19 suspected samples were sent to the laboratory for bacterial culture and ZN staining for *Mycobacterium* identification, of which 14 (73.68%) turned out to be culture-positive, resulting in the isolation of 22 organisms. The remaining samples (5, 26.32%) showed no bacterial growth. There was no *Mycobacterium* spp. identified. Among the culture positives, 50.0% (7/14) showed monomicrobial growth, and 50.0% (7/14) showed polymicrobial growth (Figure 1a). The polymicrobial growth shown varied among groups of organisms which include (1) *Klebsiella* spp. and *Enterococcus* spp.; (2) *Proteus mirabilis*, *Proteus vulgaris*, and *Providencia rettgeri*; (3) *Citrobacter* spp. and *S. aureus*; (4) *Morganella morganii* and *Proteus mirabilis*; (5) *Citrobacter diversus* and *Enterobacter* spp.; (6) *Klebsiella* spp. and *P. aeruginosa*; and (7) *Klebsiella* spp. and *Proteus mirabilis*. Among the organisms isolated, 86.36% (19/22) were Gram-negative bacilli, out of which 13 (68.42%) were Enterobacterales and 6 (31.58%) were nonfermenters, 5 (83.33%) *P. aeruginosa* and 1 (16.67%) *Pseudomonas* spp. *P. aeruginosa* (5, 22.73%) was the most common organism isolated, followed by *Klebsiella* spp. (4, 18.18%) (Figure 1b). Among the 3 (13.64%) Gram-positive isolates, 2 (66.67%) were *S. aureus* and 1 (33.33%) *Enterococci* spp. (Figure 1c). Among the Gram-negatives, AMR was predominantly associated with *Proteus vulgaris*, *Citrobacter* spp., *Klebsiella* spp., *Providencia rettgeri*, and *Morganella morganii* (Figure 1d). In the Gram-positives, *S. aureus* and *Enterococcus* spp. were resistant to tetracycline and doxycycline. Additionally, *S. aureus* was resistant to penicillin and *Enterococcus* spp. to ciprofloxacin (Figure 1e).

### 3.3. Distribution of ES*β*L Variants Among Enterobacterales Associated With SSI Post-TOS

A total of 13 (59.1%) Enterobacterales were isolated during the study period. Phenotypic ES*β*L screening and molecular characterization were investigated to identify possible resistant gene expression. Three isolates (two *Klebsiella* spp. and one *Proteus vulgaris*) were identified as ES*β*L-positive using both phenotypic screening and molecular characterization. On the contrary, the remaining 10 Enterobacterales tested ES*β*L-negative to both the phenotypic and molecular methods (Table 1).

### 3.4. Molecular Characterization of Targeted ES*β*L Genes

**Figure 2:**
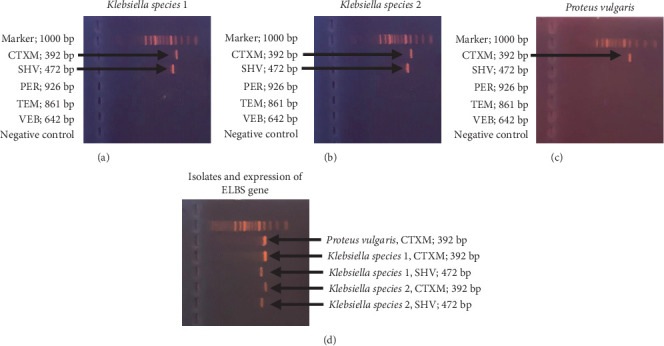
Molecular characterization of the ES*β*L genes by PCR. (a) *Klebsiella* species 1 and the ES*β*L genes expressed (CTX M, 392 bp; SHV, 472 bp). (b) *Klebsiella* species 2 and the ES*β*L genes expressed (CTX M, 392 bp; SHV, 472 bp). (c) *Proteus vulgaris* and the gene expressed (CTX M, 392 bp). (d) The isolates, genes expressed, and their respective base pair. *Klebsiella* species 1 was resistant to ciprofloxacin, levofloxacin, cefuroxime, ceftazidime, cefotaxime, and cefepime but was susceptible to meropenem, amikacin, and gentamicin. *Klebsiella* species 2 also expressed similar resistant patterns to those of *Klebsiella* species 1. *Proteus vulgaris* was resistant to ciprofloxacin, levofloxacin, gentamicin, cefuroxime, ceftazidime, cefotaxime, and cefepime but was susceptible to meropenem and amikacin.

This study considered five different ES*β*L genes for purposes of antimicrobial-resistant identification. The various genes considered, their sequences, and their respective product lengths (base pair) are represented in Table 2. The genomic DNA of all the Enterobacterales isolates was tested for the presence of ES*β*L genes using conventional PCR and agarose gel electrophoresis methods. Two genes (CTXM and SHV) were identified in two *Klebsiella* spp. (Figure 2a,b). The CTXM gene was also identified in one *Proteus vulgaris* (Figure 2c). Other genes tested, such as PER, TEM, and VEB, were not identified in any of the bacterial isolates. The three isolates that expressed the ES*β*L genes (Figure 2d) were all phenotypically ES*β*L positive (Table 1).

### 3.5. Chi-Square or Fisher's Exact Test Between Mono- and Polymicrobial Infections

Chi-square/Fisher exact analysis was performed to assess the associations between mono- and polymicrobial infections. However, there was a significant association between mono- and polymicrobial infections (*p* value < 0.001) (Table 3).

## 4. Discussion

The present study was carried out in 210 patients who underwent various orthopedic surgeries. Nineteen (19) samples were taken from patients who were suspected to have developed SSI, and out of which 14 (73.68%) cases yielded positive culture, while 5 (26.32%) cases showed no bacterial growth. The SSI incidence following TOS was 6.7%. For global context, a systematic review in *BMC Infectious Diseases* reported a pooled orthopedic SSI incidence of 5.5% globally but up to 14.6% in LMICs, validating our findings [20].

The 73.68% culture positivity observed is comparable to 68.8% in Uganda [21], Ethiopia (71%) [22], and Nepal (60.6%) [23]. This difference may reflect variations in surgical techniques, preoperative and intraoperative practices, patient-related factors, environmental factors, and postoperative care. The proportion of culture positivity in our study is comparatively lower than 90% noticed in Tanzania [24] and 96% observed in India [25]. Variations in culture positivity rates can generally be explained by a combination of factors, including differences in infection prevention and control measures and variations in patient demographics, such as age, sex, and underlying health conditions. Additionally, the timing of sample collection and differences in wound management protocols may influence the likelihood of bacterial growth in cultures. These variables highlight the importance of standardized procedures and context-specific considerations when comparing microbiological findings across studies.

*P. aeruginosa* (5, 22.73%) was the most common organism isolated, followed by *Klebsiella* spp. (4, 18.18%) and *S. aureus* (3, 13.04%). These findings align with trends in East Africa and India, where *Pseudomonas* and *Klebsiella* spp. dominate trauma-associated SSIs [24, 26].

In addition, a previous study also reported *P. aeruginosa* (9, 39.13%) as the most common organism isolated, followed by *Klebsiella* spp. (5, 21.73%) [27]. These results are in contrast with studies in which coagulase-negative staphylococci were the most common infectious microorganisms [28, 29], and in others, *S. aureus* was common [30] Among other reasons, the isolated species could vary due to differences in aseptic techniques followed, diverse geographical epidemiology of causative agents, and differences in the surgical procedures performed. Although few *S. aureus* were isolated in this study, their MRSA status was not established.

Enterobacterales isolated in this study were highly resistant to ciprofloxacin and cefuroxime (53.85% and 61.54%), respectively. These findings are in line with a study that reported similar MDR profiles in Gram-negatives from Indian trauma centers [31]. On the other hand, all the Enterobacterales were highly susceptible to gentamicin, amikacin, and meropenem (69.23%, 76.92%, and 92.31%), respectively. *P. aeruginosa* and the other *Pseudomonas* spp. identified in this study had 100% susceptibility. *S. aureus* was 100% resistant to tetracycline, doxycycline, and penicillin for the Gram-positive organisms. *Enterococcus* spp. was also 100% resistant to tetracycline, doxycycline, and ciprofloxacin. On the other hand, *Enterococcus* spp. was susceptible to penicillin (100%), but *S. aureus* was also susceptible to erythromycin, gentamicin, clindamycin, and trimethoprim/sulfamethoxazole (100% for all). Aminoglycosides and carbapenems may be preferred in ESBL infections because they bypass the primary resistance mechanism (ESBLs), offer potent bactericidal activity, and improve clinical outcomes, especially in severe or high-risk infections.

Most of the patients developed the infection during the hospital stay (after some months of admission to the hospital) (85.71%, 12/14). This could be due to exposure to the hospital environment and postoperative care. Microorganisms present in hospital settings are frequently subjected to a wide range of antimicrobial agents, which create selective pressure that favors the survival and proliferation of resistant strains. This environment accelerates the emergence of AMR, as susceptible bacteria are eliminated while resistant ones persist and multiply. Over time, this dynamic contributes to developing and maintaining multidrug-resistant organisms within healthcare facilities [32, 33], posing difficulty in therapeutic management of SSIs involving such isolates. Resistance to the commonly used antibiotics, especially cefuroxime (61.54%), could also be attributed to abuse resulting from self-medication [34]. The study highlights the need for strict measures on antimicrobial stewardship and the quest for novel antibiotics, as the relatively high level of antibiotic resistance suggests that the present antibiotics may become ineffective if prompt action is not taken.

According to recent studies, ES*β*L-producing bacteria are causing problems in a large number of healthcare facilities [35, 36]. Pathogens that produce ES*β*Ls are of serious public health concern and are challenging to treat. Such resistant pathogenic infections have the potential to turn nonfatal cases into deadly conditions due to treatment failure [37, 38]. Bacterial chromosomes include ES*β*L-coding genes, which can be passed down by heredity or transferred by plasmids within the bacterial population [39], suggesting the perpetuity of these resistant strains.

In this study, ES*β*L production (CTXM and SHV genes) was seen among three (23.08%) isolates of the Enterobacterales family, identified in two *Klebsiella* spp., and the CTXM gene in one *Proteus vulgaris*. Comparatively, the 23.08% ES*β*L rate here is lower than rates reported in similar Ghanaian studies [40, 41]. In addition, a previous study reported *CTXM* as the most frequently detected gene producing the ES*β*L phenotype [42]. The CTX-M subgroup CTX-M-15 has been reported to dominate the West African ESBL genotypes [43, 44], which the current study did not cover. In contrast, studies have reported that most ES*β*L bacteria include *E. coli* (25.9%), *Klebsiella pneumoniae* (7%), *Acinetobacter* spp. (2.4%), *P. aeruginosa* (2.4%), and *Proteus* spp. (1.2%) [31]. According to Kasukurthy and Bathala, Gram-negative bacilli are the predominant pathogens of SSIs, and *K. pneumoniae* (29%) and *E. coli* (22%) were common, and ES*β*L prevalence (44) [45] was comparatively higher than our observation.

## 5. Conclusion

Gram-negative bacteria (86.36%) were the dominant isolates from surgical sites in the study area. Among the Gram-negative bacilli, *P. aeruginosa*, 5(26.32%), followed by *Klebsiella* spp. (4, 21.05%), were the most common bacteria causing TOS-associated SSI in Northern Ghana. In Gram-positive bacteria, *S*. *aureus* 2(66.67%) were the dominant isolates from surgical sites. ES*β*L production was seen among 3(23.08%) isolates of the Enterobacterales family (CTXM and SHV genes) and was identified in two *Klebsiella* spp. each, and the CTXM gene was also identified in one *Proteus vulgaris*. The current study has revealed varied resistance of AMR with CTXM and SHV as common ES*β*L genes among the isolates. An expanded study to involve other forms of surgery will reveal a vivid scope of the AMR and ES*β*L distribution in Northern Ghana. An expanded study is needed to ensure sustainable AMR monitoring at the national and subregional levels to feed into global AMR surveillance and networks, especially considering Ghana's participation in the WHO GLASS platform. Also, a combination disc testing with cefotaxime ± clavulanate for increased sensitivity, as endorsed by CLSI and EUCAST, will facilitate easy and quicker phenotypic ES*β*L screening. Finally, further studies should consider including the multiple antibiotic resistance (MAR) index as it is widely used for environmental/clinical isolates and would strengthen AMR surveillance output.

## 6. Limitations of the Study

The relatively small sample size could influence the current findings, particularly in orthopedic surgeries, as this tends to increase the potential for bias and may not represent the broader patient population or surgical practices. Also, the absence of data on the surgeons' experience could influence the recorded incidence, as varying levels of experience may have different proficiency levels in performing procedures, leading to inconsistent surgical outcomes.

## Figures and Tables

**Table 1:** Distribution of ES*β*L variants among Enterobacterales involved in SSIs post TOS.

		**Screening method**	
		**Phenotypic (ES*β*L positive)**	**PCR (ES*β*L positive)**
**Isolates**	**Total no.**	**N** ** (%)**	**N** ** (%)**
*Klebsiella* spp.	4	2 (50.0)	2 (50.0)
*Citrobacter diversus*	1	0 (0.0)	0 (0.0)
*Citrobacter* spp.	1	0 (0.0)	0 (0.0)
*Enterobacter* spp.	1	0 (0.0)	0 (0.0)
*Proteus mirabilis*	3	0 (0.0)	0 (0.0)
*Proteus vulgaris*	1	1 (100.0)	1 (100.0)
*Morganella morganii*	1	0 (0.0)	0 (0.0)
*Providencia rettgeri*	1	0 (0.0)	0 (0.0)

**Table 2:** Target genes screened for purposes of ESBL.

**Genes**	**Sequence**	**Product length**	**Purpose**
PER-F	ATGAATGTTCATTATAAAAGC	926	ES*β*L
PER-R	TTAATTTGGGCTTAGGG		ES*β*L
CTX M-F	GCGATGGGCAGTACCAGTAA	392	ES*β*L
CTX M-R	TTACCCAGCGTCAGATTCCG		ES*β*L
SHV-F	TCAGCGAAAAACACCTTG	472	ES*β*L
SHV-R	TCCCGCAGATAAATCACCA		ES*β*L
TEM-F	ATGAGTATTCAACATTTCCG	861	ES*β*L
TEM-R	TTACCAATGCTTAATCAGTGAG		ES*β*L
VEB-F	CGACTTCCATTTCCCGATGC	642	ES*β*L
VEB-R	GGACTCTGCAACAAATACGC		ES*β*L

**Table 3:** Chi-square or Fisher's exact test between mono- and polymicrobial infections.

**Variables**	**Developed a surgical site infection?**			**p** ** value**
	**No (196)**	**Yes (14)**	**Total (210)**	**< 0.001**
No infection	196 (100.0)	0 (0.0)	196 (93.3)	
Monomicrobial infection	0 (0.0)	7 (50.0)	7 (3.3)	
Polymicrobial infection	0 (0.0)	7 (50.0)	7 (3.3)	

## Data Availability

The data that support the findings of this study are available from the corresponding author upon reasonable request.

## References

[1] WHO (2016). *Global Guidelines for the Prevention of Surgical Site Infection*.

[2] Sarangi S. K., Padhi S. (2019). Bacteriological Profile of Post-Operative Orthopedic Implant Infections and Their Antibiotic Sensitivity Pattern in a Tertiary Care Hospital of Southern Odisha. *Journal of Dr NTR University of Health Sciences*.

[3] Najjar Y. W., Saleh M. Y. (2017). Orthopedic Surgical Site Infection: Incidence, Predisposing Factors, and Prevention. *International Journal Of Medical Science And Clinical Invention*.

[4] Bhat A. K., Parikh N. K., Acharya A. (2018). Orthopaedic Surgical Site Infections: A Prospective Cohort Study. *Canadian Journal of Infection Control*.

[5] Lakshminarayana S., Chavan S., Prakash R., Sangeetha S. (2013). Bacteriological Profile of Orthopedic Patients in a Tertiary Care Hospital, Bengaluru. *International Journal of Science and Research*.

[6] Allegranzi B., Aiken A. M., Kubilay N. Z. (2018). A Multimodal Infection Control and Patient Safety Intervention to Reduce Surgical Site Infections in Africa: A Multicentre, Before–After, Cohort Study. *Lancet Infectious Diseases*.

[7] Al-Mulhim F. A., Baragbah M. A., Sadat-Ali M., Alomran A. S., Azam M. Q. (2014). Prevalence of Surgical Site Infection in Orthopedic Surgery: A 5-Year Analysis. *International Surgery*.

[8] Vikesland P., Garner E., Gupta S., Kang S., Maile-Moskowitz A., Zhu N. (2019). Differential Drivers of Antimicrobial Resistance Across the World. *Accounts of Chemical Research*.

[9] Birgand G., Castro-Sánchez E., Hansen S. (2018). Comparison of Governance Approaches for the Control of Antimicrobial Resistance: Analysis of Three European Countries. *Antimicrobial Resistance & Infection Control*.

[10] Ibrahim D. R., Dodd C. E., Stekel D. J., Ramsden S. J., Hobman J. L. (2016). Multidrug Resistant, Extended Spectrum *Β*-Lactamase (ESBL)-Producing Escherichia coli Isolated From a Dairy Farm. *FEMS Microbiology Ecology*.

[11] Dyatlov I., Astashkin E., Kartsev N., Mendez-Vilas A. (2015). Novel blaCTX-M-2-Type Gene Coding Extended Spectrum Beta-Lactamase CTX-M-115 Discovered in Nosocomial Acinetobacter baumannii Isolates in Russia. *Multidisciplinary Approaches for Studying and Combating Microbial Pathogens*.

[12] Nagshetty K., Shilpa B., Patil S. A., Shivannavar C., Manjula N. (2021). An Overview of Extended Spectrum Beta Lactamases and Metallo Beta Lactamases. *Advances in Microbiology*.

[13] Viana Marques D. A., Machado S. E. F., Ebinuma V. C. S., Duarte C. A. L., Converti A., Porto A. L. F. (2018). Production of *β*-Lactamase Inhibitors by Streptomyces Species. *Antibiotics*.

[14] Labi A. K., Bjerrum S., Enweronu-Laryea C. C. (2020). High Carriage Rates of Multidrug-Resistant Gram-Negative Bacteria in Neonatal Intensive Care Units From Ghana. *Open Forum Infectious Diseases*.

[15] Osei K. M., Choi H., Zeyeh D. E. (2022). Multidrug-Resistant Gram-Negative Bacterial Infections the Tamale Teaching Hospital in Northern Ghana: A Three-Year Retrospective Analysis. *Annals of Clinical Microbiology*.

[16] Dela H., Egyir B., Majekodunmi A. O. (2022). *Diarrhoeagenic* E. coli Occurrence and Antimicrobial Resistance of Extended Spectrum Beta-Lactamases Isolated From Diarrhoea Patients Attending Health Facilities in Accra, Ghana. *PLoS One*.

[17] van den Brink R. (2021). *The Beginning of the End. The End of an Antibiotic Era: Bacteria’s Triumph Over a Universal Remedy*.

[18] Bhatt N. R., Murphy C. A., Wall N. (2020). Implications of Faecal ESBL Carriers Undergoing TRUS-Guided Prostate Biopsy (TRUSPB): Role of Screening Prior to TRUSPB. *Irish Journal of Medical Science*.

[19] Centers for Disease Control and Prevention (2013). CDC/NHSN Protocol Corrections, Clarification, and Additions. https://www.socinorte.com/wp-content/uploads/2013/03/Criterios-de-IN-2013.pdf.

[20] Glasbey J. C. (2023). *Developing a Pathway for Remote Assessment of Surgical Wounds With Partners in Low-and Middle-Income Countries: An Approach for Efficient Trials and Resilient Perioperative Systems*.

[21] Seni J., Najjuka C. F., Kateete D. P. (2013). Antimicrobial Resistance in Hospitalized Surgical Patients: A Silently Emerging Public Health Concern in Uganda. *BMC Research Notes*.

[22] Starčević S., Munitlak S., Mijović B., Mikić D., Šuljagić V. (2015). Surgical Site Infection Surveillance in Orthopedic Patients in the Military Medical Academy, Belgrade. *Vojnosanitetski Pregled*.

[23] Amatya J., Rijal M., Baidya R. (2015). Bacteriological Study of the Postoperative Wound Samples and Antibiotic Susceptibility Pattern of the Isolates in BB Hospital. *JSM Microbiology*.

[24] Manyahi J., Matee M. I., Majigo M., Moyo S., Mshana S. E., Lyamuya E. F. (2014). Predominance of Multi-Drug Resistant Bacterial Pathogens Causing Surgical Site Infections in Muhimbili National Hospital, Tanzania. *BMC Research Notes*.

[25] Rao R., Sumathi S., Anuradha K., Venkatesh D., Krishna S. (2013). Bacteriology of Postoperative Wound Infections. *International Journal Of Pharmaceutical And Bio-Medical Science*.

[26] Chen A. F., Wessel C. B., Rao N. (2013). Staphylococcus aureus Screening and Decolonization in Orthopaedic Surgery and Reduction of Surgical Site Infections. *Clinical Orthopaedics and Related Research*.

[27] Meng J., Zhu Y., Li Y. (2020). Incidence and Risk Factors for Surgical Site Infection Following Elective Foot and Ankle Surgery: A Retrospective Study. *Journal of Orthopaedic Surgery and Research*.

[28] Li G.-q., Guo F.-f., Ou Y., Dong G.-w., Zhou W. (2013). Epidemiology and Outcomes of Surgical Site Infections Following Orthopedic Surgery. *American Journal of Infection Control*.

[29] Becker K., Both A., Weißelberg S., Heilmann C., Rohde H. (2020). Emergence of Coagulase-Negative Staphylococci. *Expert Review of Anti-Infective Therapy*.

[30] Shafizad M., Shafiee S., Ebrahimzadeh K., Ehteshami S., Haddadi K., Abedi M. (2019). Effect of Topical Vancomycin on Prevention of Surgical Site Infection in Spinal Surgery. *Journal of Mazandaran University of Medical Sciences*.

[31] Madhavi R. B., Hanumanthappa A. (2021). Prevalence of Extended Spectrum Beta-Lactamase Producing Gram-Negative Bacilli Causing Surgical Site Infections in a Tertiary Care Centre. *Journal of Pure and Applied Microbiology*.

[32] Sievert D. M., Ricks P., Edwards J. R. (2013). Antimicrobial-Resistant Pathogens Associated With Healthcare-Associated Infections: Summary of Data Reported to the National Healthcare Safety Network at the Centers for Disease Control and Prevention, 2009-2010. *Infection Control & Hospital Epidemiology*.

[33] Rothe C., Schlaich C., Thompson S. (2013). Healthcare-Associated Infections in Sub-Saharan Africa. *Journal of Hospital Infection*.

[34] Al Meslamani A. Z. (2023). Antibiotic Resistance in Low-and middle-Income Countries: Current Practices and Its Global Implications. *Expert Review of Anti-infective Therapy*.

[35] Mohammed I., Abass E. (2019). Phenotypic Detection of Extended Spectrum *β*-Lactamases (ESBL) Among Gram Negative Uropathogens Reveals Highly Susceptibility to Imipenem. *Pakistan Journal of Medical Sciences*.

[36] Tansarli G. S., Athanasiou S., Falagas M. E. (2013). Evaluation of Antimicrobial Susceptibility of Enterobacteriaceae Causing Urinary Tract Infections in Africa. *Antimicrobial Agents and Chemotherapy*.

[37] Aruhomukama D. (2020). Review of Phenotypic Assays for Detection of Extended-Spectrum *β*-Lactamases and Carbapenemases: A Microbiology Laboratory Bench Guide. *African Health Sciences*.

[38] Shan S., Sajid S., Ahmad K. (2015). Detection of Bla IMP Gene in Metallo-*β*-Lactamase Producing Isolates of Imipenem Resistant Pseudomonas aeruginosa; an Alarming Threat. *Journal of Microbiological Research*.

[39] Naelasari D. N., Koendhori E. B., Dewanti L., Sarassari R., Kuntaman K. (2018). The Prevalence of Extended Spectrum Beta-Lactamase (ESBL) Producing Gut Bacterial Flora Among Patients in Dr. Soetomo Hospital and Primary Health Centre in Surabaya. *Folia Medica Indonesiana*.

[40] Sanders T., Bentum J., Fox A., Egyir B., Watters C. (2022). Characterization of MRSA and ESBL Pathogens From Patients With Surgical-Site Infections in Accra, Ghana. *Antimicrobial Stewardship & Healthcare Epidemiology*.

[41] Bediako-Bowan A. A., Kurtzhals J. A., Mølbak K., Labi A.-K., Owusu E., Newman M. J. (2020). High Rates of Multi-Drug Resistant Gram-Negative Organisms Associated With Surgical Site Infections in a Teaching Hospital in Ghana. *BMC Infectious Diseases*.

[42] Egyir B., Nkrumah-Obeng N., Nyarko E., Fox A., Letizia A., Sanders T. (2020). Prevalence of Extended Spectrum Beta-Lactamase Producing *Escherichia* coli, *Klebsiella pneumoniae* and *Pseudomonas aeruginosa* From Hospital Acquired Surgical Site Infections in Ghana. *Open Forum Infectious Diseases*.

[43] Tansarli G. S., Poulikakos P., Kapaskelis A., Falagas M. E. (2014). Proportion of Extended-Spectrum *Β*-Lactamase (ESBL)-Producing Isolates Among Enterobacteriaceae in Africa: Evaluation of the Evidence—Systematic Review. *Journal of Antimicrobial Chemotherapy*.

[44] Dela H., Egyir B., Behene E. (2023). Microbiological Quality and Antimicrobial Resistance of Bacteria Species Recovered From Ready-to-Eat Food, Water Samples, and Palm Swabs of Food Vendors in Accra, Ghana. *International Journal of Food Microbiology*.

[45] Kasukurthy L., Bathala M. (2020). Bacteriological Profile of Surgical Site Infections (SSIs)-a Study in a Tertiary Care Hospital. *Journal of Evidence-Based Medicine and Healthcare*.

